# Epoxomicin-functionalized thermosensitive hydrogel rescues bone defects in osteoporosis by the NF-κB signaling pathway

**DOI:** 10.1093/rb/rbag141

**Published:** 2026-07-15

**Authors:** Liqun Jiang, Qijia Sui, Chen Sun, Chen Li, Shuai Wang, Zijiao Zhang, Huiying Liu, Guowu Ma

**Affiliations:** School of Stomatology, Dalian Medical University, Dalian 116044, China; Academician Laboratory of Immune and Oral Development & Regeneration, Dalian Medical University, Dalian 116044, China; School and Hospital of Stomatology, Dalian Medical University, Dalian 116044, China; School of Stomatology, Dalian Medical University, Dalian 116044, China; Academician Laboratory of Immune and Oral Development & Regeneration, Dalian Medical University, Dalian 116044, China; School and Hospital of Stomatology, Dalian Medical University, Dalian 116044, China; School of Stomatology, Dalian Medical University, Dalian 116044, China; Academician Laboratory of Immune and Oral Development & Regeneration, Dalian Medical University, Dalian 116044, China; School and Hospital of Stomatology, Dalian Medical University, Dalian 116044, China; School of Stomatology, Dalian Medical University, Dalian 116044, China; Academician Laboratory of Immune and Oral Development & Regeneration, Dalian Medical University, Dalian 116044, China; School and Hospital of Stomatology, Dalian Medical University, Dalian 116044, China; School of Stomatology, Dalian Medical University, Dalian 116044, China; Academician Laboratory of Immune and Oral Development & Regeneration, Dalian Medical University, Dalian 116044, China; School and Hospital of Stomatology, Dalian Medical University, Dalian 116044, China; School of Stomatology, Dalian Medical University, Dalian 116044, China; Academician Laboratory of Immune and Oral Development & Regeneration, Dalian Medical University, Dalian 116044, China; School and Hospital of Stomatology, Dalian Medical University, Dalian 116044, China; School of Stomatology, Dalian Medical University, Dalian 116044, China; Academician Laboratory of Immune and Oral Development & Regeneration, Dalian Medical University, Dalian 116044, China; School and Hospital of Stomatology, Dalian Medical University, Dalian 116044, China; School of Stomatology, Dalian Medical University, Dalian 116044, China; Academician Laboratory of Immune and Oral Development & Regeneration, Dalian Medical University, Dalian 116044, China; School and Hospital of Stomatology, Dalian Medical University, Dalian 116044, China

**Keywords:** epoxomicin, smart hydrogel, osteoporosis, inflammation, osteoclast differentiation

## Abstract

To address the clinical challenge of difficult bone defect healing in osteoporosis patients, this study developed a functional delivery system based on a thermosensitive hydrogel loaded with epoxomicin (CS-βGP@10Epoxomicin). This system possesses injectability and body-temperature-triggered *in situ* gelation properties, overcoming the poor targeting and potential complications associated with systemic drug administration, while achieving dual anti-inflammatory and osteoclast-inhibitory effects within the local bone defect microenvironment. *In vitro* experiments confirmed its ability to significantly reduce the expression of inflammatory cytokines and osteoclast-related factors. In an ovariectomized (OVX) mouse femoral defect model, local application of this hydrogel significantly increased bone mineral density (BMD) at the defect site by 53.47% compared to the OVX group and downregulated the expression of the inflammatory cytokine interleukin-1 beta (IL-1β) by 27.37%. The system maintained favorable biocompatibility while effectively inhibiting the activation of the nuclear factor kappa-B (NF-κB) signaling pathway, alleviating inflammatory responses and suppressing osteoclast differentiation. Transcriptomic analysis of a public dataset further supported the central role of the NF-κB pathway in the pathological microenvironment of osteoporosis. In summary, this study establishes an intelligent localized delivery strategy that integrates targetability, safety and therapeutic efficacy, providing a potential solution for the local treatment of osteoporotic bone defects.

## Graphical abstract

**Graphical Abstract:**
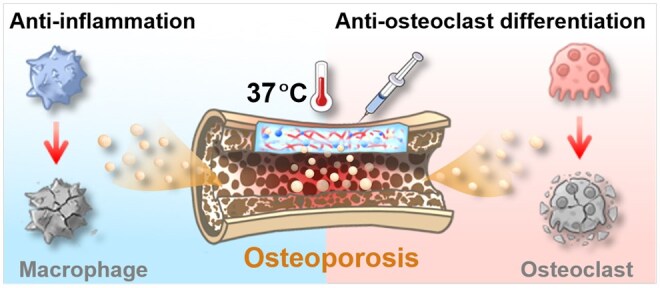

## Introduction

Osteoporosis is a systemic bone disease characterized by reduced bone mass and microstructural damage, leading to increased bone fragility and fracture risk [1]. It has become a global public health issue [2]. Its pathological microenvironment manifests as an imbalance in bone homeostasis, characterized by excessive osteoclast activity and an accompanying inflammatory state [3]. This imbalance not only accelerates bone loss but also involves the secretion of inflammatory factors such as tumor necrosis factor-alpha (TNF-α) and interleukin-6 (IL-6), which inhibit bone repair [4]. This process increases the incidence of fractures and impairs bone healing after injury, often leading to delayed union or nonunion [5]. In this process, the nuclear factor kappa-B (NF-κB) signaling pathway serves as a central hub regulating osteoclast differentiation and inflammatory responses, playing a crucial role in the pathological progression of osteoporosis [6]. On one hand, this pathway can be activated by factors such as the receptor activator of NF-κB ligand (RANKL), inducing the expression of transcription factors like nuclear factor of activated T-cells 1 (NFATc1), thereby controlling osteoclast differentiation and bone resorption [6]. On the other hand, under inflammatory conditions, NF-κB activation promotes the release of pro-inflammatory cytokines, exacerbating bone destruction driven by osteoclast differentiation [7]. Therefore, precise modulation of NF-κB activity within the bone microenvironment, aimed at inhibiting osteoclast differentiation and inflammatory responses, represents a promising therapeutic strategy for the repair of osteoporotic bone defects [8].

Epoxomicin, a highly specific proteasome inhibitor, regulates the activity of the NF-κB signaling pathway, thereby reducing inflammatory reactions and inhibiting the differentiation of osteoclasts [9, 10]. In terms of anti-inflammatory effect, epoxomicin demonstrates significant anti-inflammatory activity *in vivo* by effectively inhibiting the activation of NF-κB, thereby blocking the inflammatory response in the murine ear edema model [9]. As for the inhibition of osteoclast differentiation, its mechanism is also closely related to the NF-κB pathway [11]. Studies have shown that epoxomicin inhibits the degradation of inhibitor of nuclear factor κBα (IκBα) protein, blocking the activation of the NF-κB signaling pathway, which suppresses osteoclast differentiation [10]. Among proteasome inhibitors with comparable inhibition potential, epoxomicin requires lower concentrations to achieve an equivalent inhibition effect, indicating higher potency [10]. Epoxomicin is therefore an ideal candidate for targeting the NF-κB pathway to interfere with the pathological process of osteoporotic bone defects. However, systemic administration of drugs suffers from problems such as poor targeting and significant systemic toxicity, which limits its clinical application [12]. Therefore, the development of biomaterial-based drug delivery systems capable of achieving localized and precise drug administration is of paramount importance for enhancing therapeutic efficacy and reducing toxic side effects [13].

The chitosan–β-glycerophosphate (CS-βGP) thermosensitive hydrogel system demonstrates comprehensive advantages in the fields of tissue engineering and drug delivery [14]. This system is composed of the natural polysaccharide CS and the βGP [15]. Its intelligent thermosensitive property enables rapid gelation upon injection into the body [16]. This allows it to perfectly conform to and fill various irregular tissue defects through minimally invasive injection, effectively addressing the issue of poor matching associated with the implantation of traditional pre-formed scaffolds [16]. The characteristic of *in situ* gelation enables the localized release of drugs at the lesion site [17]. This ensures effective therapeutic drug concentrations locally while significantly reducing systemic exposure to the drug, thereby minimizing systemic risks [18]. This advantage is particularly important in treating local infections, tumors or tissue defects [14, 19]. For instance, in the treatment of bone infections, the local sustained release of antibiotics can effectively overcome the limited efficacy of systemic administration caused by poor blood supply [20]. Compared to traditional biomaterials such as poly(lactic-co-glycolic acid) (PLGA) microspheres or collagen sponges, the CS-βGP system possesses a three-dimensional (3D) porous structure and high water content that more closely resemble the natural extracellular matrix, providing an ideal microenvironment for cell adhesion, proliferation and differentiation [21, 22]. In contrast to some natural hydrogels with weaker mechanical strength (such as pure alginate or hyaluronic acid hydrogels), the CS-βGP system forms a more stable network structure with a certain degree of mechanical strength, thereby better simulating the mechanical properties of tissues [19, 23]. Therefore, the CS-βGP thermosensitive hydrogel system, with its excellent biocompatibility, injectability and *in situ* phase transition capability, provides an ideal platform for localized drug delivery [24].

**Figure 1:**
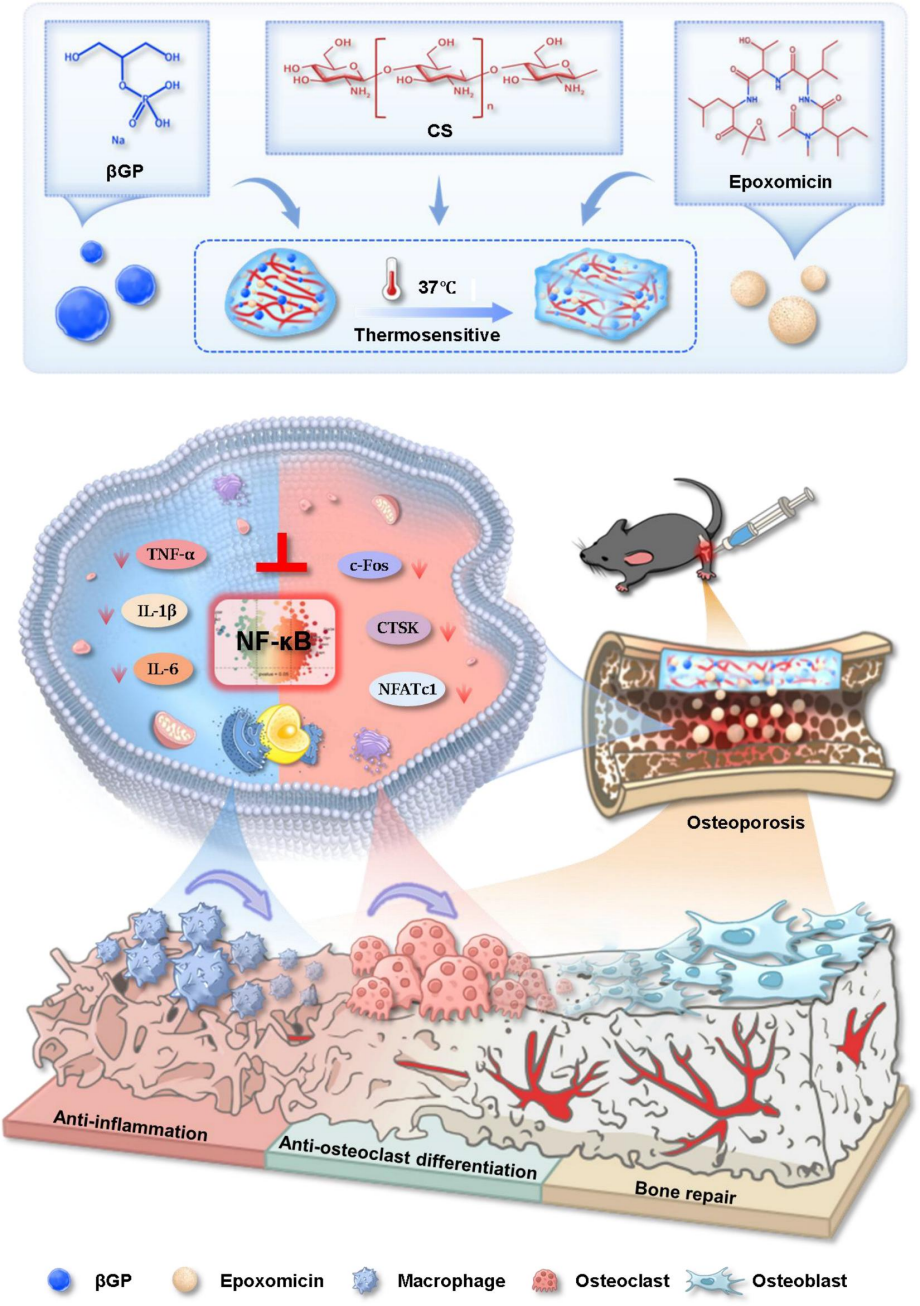
Schematic diagram of the preparation of CS-βGP@Epoxomicin and its therapeutic mechanism in osteoporosis.

This study developed a functionalized epoxomicin delivery system based on thermosensitive hydrogels. This system retains the advantages of thermosensitive hydrogels such as high biocompatibility, injectability and *in situ* gelling ability, achieving localized drug release and precise regulation at the bone defect site, thereby reducing systemic toxic side effects. This delivery system can effectively inhibit the activation of the NF-κB signaling pathway, reduce the secretion of inflammatory factors and inhibit the differentiation of osteoclasts, thereby repairing osteoporotic bone defects. This strategy provides a new intelligent therapeutic approach to repair osteoporotic bone defects by integrating targeting, safety and high efficiency (Figure 1).

## Materials and methods

### Synthesis of epoxomicin-CS-βGP disodium hydrogel (CS-βGP@Epoxomicin)

Four hundred milligrams of chitosan powder (degree of deacetylation ≥95%, viscosity-average molecular weight 200–400 kDa, Macklin, Shanghai, China) were completely dissolved in 16 mL of 0.1 M acetic acid (Sinopharm, Shanghai, China) to obtain a 2.5% (w/v) chitosan solution. The powder of epoxomicin (Aladdin, Shanghai, China) was first dissolved in dimethyl sulfoxide (DMSO, Solarbio, Beijing, China) to prepare a concentrated stock solution, which was then diluted with deionized water (DI water) to prepare a 5 µM working solution with a final DMSO concentration below 0.1% (v/v). Then the solution was mixed with 1.3 M sterile βGP disodium (Coolaber, Beijing, China) solution and 2.5% (w/v) chitosan solution to obtain epoxomicin hydrogels with final concentrations of 1, 5, 10 and 20 nM. After incubation at 37°C for 1 h, these solutions underwent a transformation from sol to gel, forming stable colloidal gels.

### Materials characterization

#### Flowability

CS-βGP sol was slowly poured into a pentagonal mold with a diameter of 55 mm and a thickness of 2 mm at a constant speed along with the sol loaded with 5 nM and 10 nM epoxomicin. Three minutes later, the gel was photographed and its area was measured using ImageJ software (National Institutes of Health, USA).

#### Injectability

The injection force versus displacement was measured for both materials using two different needle types (22 G × 25 mm, 25 G × 16 mm) to simulate various clinical scenarios.

#### Gelation temperature

Glass bottles containing CS-βGP and CS-βGP@Epoxomicin sols were incubated at 37°C to determine the precise kinetics of the sol–gel transformation. Observations were conducted at granular intervals of 5, 10, 15, 30 and 60 min. Then, the gel state is evaluated by inverting the bottles 180° and observing the fluidity of the contents.

#### Scanning electron microscope

CS-βGP and CS-βGP@Epoxomicin hydrogels were dried in vacuum freeze dryer at −80°C (scientz-10 NA, Ningbo, China) for 48 h. Then the freeze-dried samples were cut into blocks of 2 cm × 2 cm and sputtered with a conductive metal layer. Subsequently, SEM images were randomly captured using a scanning electron microscope (SEM, IT800-SHL, Jeol, Tokyo, Japan) at magnifications of 500×.

#### Swelling capacity

The swelling capacity of the hydrogel was determined by gravimetric method. Two milliliters of the hydrogel precursor solution were gelated with various concentrations of epoxomicin (0, 1, 5, 10 and 20 nM) and then lyophilized to a constant weight (*W*_1_). The dried samples were soaked in simulated body fluids (SBF, Coolaber, Beijing, China) at 37°C for 24 h. After incubation, the sample was removed, the surface moisture was absorbed and the swelling weight (*W*_2_) was recorded. The calculation formula for the expansion ratio (SR) is:


$$\text{SR}\;(\%)=[({\text{W}}_{2}-{\text{W}}_{1})/{\text{W}}_{1}]\times 100\%.$$

#### Fourier transform infrared

The chemical structure and potential interactions of the freeze-dried hydrogel samples were characterized using the VERTEX70 (Bruker Corporation, Germany) Fourier transform infrared (FTIR) spectrometer. The spectra were recorded in the range of 4000–500 cm^−1^ with a resolution of 4 cm^−1^, and each sample was scanned 32 times. All spectra were baseline corrected and analyzed to determine the characteristic functional groups and bonds.

#### Viscosity

The sample was loaded onto the fixture, the measurement gap was set to 1.0 mm and it was balanced at 37°C for 5 min. To ensure that the test is conducted within the linear viscoelastic region, the 1% strain was confirmed to be applicable for the subsequent oscillation test through strain scanning (0.1–10%, fixed frequency 10 rad s^−1^). The steady-state shear behavior of the sample was characterized by the steady-state flow test: at 37°C, the shear rate was scanned logarithmically from 0.1 to 500 s^−1^ and the changes in apparent viscosity and shear stress were recorded to evaluate its shear-thinning characteristics.

#### Compression mechanical property

The compressive mechanical properties of cylindrical hydrogel samples (diameter: 18 mm; height: 8 mm) from each group (CS-βGP and CS-βGP@Epoxomicin) were assessed using a universal testing machine. Samples were compressed at a constant rate of 2 mm/min. Stress–strain curves were generated to calculate the compressive modulus from the linear elastic region. Data were analyzed using GraphPad Prism.

#### pH stability

CS-βGP and CS-βGP@Epoxomicin (0, 1, 5, 10 and 20 nM) (1 mL) were prepared in centrifuge tubes. Subsequently, 5 mL of SBF (Coolaber, Beijing, China) was added to each sample and incubated at 37°C. At the predetermined time points (4, 6, 8, 12, 24 and 48 h), the pH value of the surrounding SBF was measured at room temperature using a calibrated pH meter.

#### Degradation capacity

The degradation characteristics of the hydrogel were evaluated *in vitro* by gravimetric method. In short, 1 mL of CS-βGP or CS-βGP@Epoxomicin solution was gelated in a pre-weighed tube at 37°C for 1 h to obtain the initial weight (*W*_1_). Then 10 mL of preheated SBF was added and gently shaken and incubated at 37°C. At the specified time point, the hydrogel was removed, the surface moisture was absorbed and the wet weight (*W*_2_) was recorded. The degradation rate is calculated as:


$$[({\text{W}}_{1}-{\text{W}}_{2})/{\text{W}}_{1}]\times 100\%.$$

### The biological effect of CS-βGP@Epoxomicin *in vitro*

#### Preparation of extract solution

CS-βGP, CS-βGP@1Epoxomicin, CS-βGP@5Epoxomicin, CS-βGP@10Epoxomicin and CS-βGP@20Epoxomicin hydrogels were soaked in α-mem medium at a ratio of 1:1 (v/v) for 24 h. The corresponding hydrogel extract was obtained. Then, 10% fetal bovine serum (FBS) and 1% penicillin/streptomycin (P/S) were added to the extract as the final extraction medium for cell culture.

#### Cell culture

Bone marrow-derived macrophages (BMMs) were isolated from the femurs and tibiae of 6-week-old C57BL/6J mice. All animal procedures were approved by the Animal Ethics Committee of Dalian Medical University, and the mice were housed in a specific pathogen-free (SPF) facility. Briefly, the femurs and tibiae were dissected and immersed in α-MEM medium (Gibco, NY, USA). The bone marrow was flushed out with α-MEM medium and treated with ACK lysis buffer (Gibco, NY, USA) to remove red blood cells. After centrifugation, the cell pellet was collected and resuspended in complete α-MEM medium supplemented with 50 ng/mL M-CSF (T&L Biotechnology, Beijing, China), 10% FBS (Gibco, NY, USA) and 1% P/S (Abbkine, Wuhan, China). The cells were then cultured at 37°C in a CO_2_ incubator (Thermo Fisher, MA, USA). After 5 days of culture, the BMMs were purified and fully differentiated.

#### Cell counting-kit

BMMs were seeded in 96-well plates at a density of 5 × 10^3^ cells per well. After the cells had adhered to the plate surface, the culture medium was replaced with extraction medium and incubated with the BMMs for 24 h. Subsequently, the cells were washed with phosphate buffered saline (PBS, Solarbio, Beijing, China) and incubated with Cell counting-kit (CCK-8) working solution (Abbkine, Wuhan, China) for 1 h at 37°C. Absorbance at 450 nm was measured using a microplate reader (Bio-Rad Laboratories, CA, USA) and cell viability was calculated based on the obtained optical density (OD) values.

#### Quantitative real-time PCR

BMMs were seeded in 6-well plates at a density of 1 × 10^5^ cells per well and cultured overnight to allow adherence. The cells were then divided into three major treatment groups: (1) control group, treated with the extract medium of either CS-βGP or CS-βGP@10Epoxomicin; (2) inflammatory group, co-treated with 100 ng/mL lipopolysaccharide (LPS, Solarbio, Beijing, China) and the extract medium of CS-βGP, CS-βGP@5Epoxomicin or CS-βGP@10Epoxomicin for 6 h and (3) osteoclastogenesis group, co-treated with 100 ng/mL RANKL (Thermo Fisher, MA, USA) and the extract medium of CS-βGP, CS-βGP@5Epoxomicin or CS-βGP@10Epoxomicin for 3 days. Following treatment, total RNA was extracted from the cells using RNAex Pro Reagent (Accurate Biotechnology, Changsha, China), following the manufacturer’s instructions. Briefly, the lysate was mixed with RNA Extraction Assisted Reagent (Accurate Biotechnology, Changsha, China) and isopropanol (Macklin, Shanghai, China), followed by washing with 75% ethanol (Macklin, Shanghai, China). The purified RNA pellet was air-dried and then dissolved in an appropriate volume of diethylpyrocarbonate (DEPC)-treated water (Coolaber, Beijing, China). Subsequently, cDNA was synthesized using the Evo M-MLV Reverse Transcription Premix Kit (Accurate Biotechnology, Changsha, China). Quantitative real-time PCR (RT-qPCR) was performed using the SYBR Green Pro Taq HS Premix (Accurate Biotechnology, Changsha, China) on a CFX96 Real-Time PCR Detection System (Bio-Rad, CA, USA). The reaction mixture contained cDNA, gene-specific primers and the premix. The mRNA expression levels of *Il-1β*, *Tnf-α*, *Ctsk* and *Nfatc1* were analyzed with *Gapdh* serving as the internal reference. The primer sequences used for amplification are listed in Table 1.

**Table 1:** Primers used for inflammatory and osteoclast differentiation-related genes in RT-qPCR.

Gene	Forward primer (5′–3′)	Reverse primer (5′–3′)
*Gapdh*	*TGTGTCCGTCGTGGATCTGA*	*TTGCTGTTGAAGTCGCAGGG*
*Tnf-α*	*CAGAAAGCATGATCCGCGAC*	*CTGCCACAAGCAGGAATGG*
*Il-1β*	*TGGTGTGTGACGTTCCCATTA*	*TCGTTGCTTGGTTCTCCTTGT*
*c-Fos*	*AGGCAGAACCCTTTGA*	*GGTGACCACGGGAGTA*
*Ctsk*	*TACCCATAT GTGGGCCAGGA*	*ATAGCCCACCACCAACACTG*

#### Western blot

The cell treatment regimen was identical to that described in Section ‘Quantitative real-time PCR’. Following treatment, total protein was extracted from the cells using cell lysis buffer (Cell Signaling Technology, MA, USA) supplemented with a phosphatase inhibitor cocktail and a protease inhibitor cocktail (Seven, Beijing, China). The lysates were centrifuged at 14 000 rpm for 15 min at 4°C, and the supernatant containing the soluble protein fraction was collected. Subsequently, equal amounts of protein were denatured by boiling at 99°C for 10 min with loading buffer (Epizyme Biotech, Shanghai, China). The denatured samples were separated by 12.5% sodium dodecyl sulfate-polyacrylamide gel electrophoresis (SDS-PAGE, Epizyme Biotech, Shanghai, China) using a Rapid High-Resolution Electrophoresis Buffer (Servebio, Wuhan, China) at a constant voltage of 200 V for approximately 1 h. The separated proteins were then transferred onto a 0.22 µm polyvinylidene difluoride (PVDF) membrane (Biosharp, Hefei, China) at a constant current of 300 mA for 30 min. Following transfer, the membranes were blocked with 5% (w/v) non-fat milk powder dissolved in tris-buffered saline containing 0.1% Tween-20 (TBST, Coolaber, Beijing, China) for 1 h at room temperature. The blocked membranes were subsequently incubated overnight at 4°C with gentle shaking in TBST and the corresponding primary antibodies: rabbit monoclonal anti-IL-1β (1:1000 dilution, HA601036, Huabio, Hangzhou, China) and mouse monoclonal anti-TNF-α (1:1000 dilution, ER65189, Huabio, Hangzhou, China). β-Actin (1:10 000 dilution, 66009-1-Ig, Proteintech, Wuhan, China) was used as an internal loading control. After three washes with TBST (10 min each), the membranes were incubated with a horseradish peroxidase (HRP)-conjugated goat anti-rabbit IgG (H + L) secondary antibody (1:10 000 dilution, SA00001-2, Wuhan, China) for 1 h at room temperature. Following washes with TBST, the protein bands were visualized using an enhanced chemiluminescence (ECL) substrate kit (Abkkine, Wuhan, China) and imaged with a ChemiDoc™ Imaging System (Bio-Rad, USA). Protein expression levels were quantified using ImageJ software.

#### Immunofluorescence staining

BMMs were co-treated with 1 μM BAY 11-7082 (MCE, Shanghai, China) and the extract medium from CS-βGP@10Epoxomicin and LPS for 6 h. Upon reaching 80–90% confluence, the cells were washed with PBS and fixed with 4% (v/v) paraformaldehyde (PFA) for 20 min, followed by permeabilization with 0.5% Triton X-100 for 20 min and three washes with PBS. The cells were blocked with goat serum at room temperature for 1 h, and then incubated with anti-p65 primary antibody (1:200, ET1603-12, Hangzhou, Huabio, China) and anti-IκBα primary antibody (1:200, 18220-1-AP, Proteintech, Wuhan, China) at room temperature overnight. After primary antibody incubation, the cells were incubated with secondary antibody (1:100) at room temperature for 1 h in the dark. All cells were washed three times with PBS and mounted with antifade mounting medium. Final images were captured using a fluorescence microscope.

### The biological effect of CS-βGP@Epoxomicin *in vivo*

#### Animal models

Six-week-old female C57BL/6J mice were purchased from Changsheng Biotechnology Co., Ltd. (Liaoning, China) and housed in the SPF animal facility of Dalian Medical University under standard conditions (12-h light/dark cycle, 22 ± 2°C, 50–60% humidity) with free access to food and water. All animal experiments were approved by the Animal Ethics Committee of Dalian Medical University and were performed in accordance with institutional guidelines for animal care and use. The approval number was XL251010475.

After 1 week of adaptation, 20 mice were randomly divided into two initial surgery groups: the Sham operation group (SHAM) (*n* = 5) and the ovariectomy group (OVX) (*n* = 15). All mice were anesthetized by intraperitoneal injection of tribromoethanol (Aladdin, Shanghai, China). The SHAM group exposed the ovaries but did not remove them. For the OVX group, bilateral ovariectomy was performed through a dorsolateral incision. After a 3-week recovery period, a second operation was performed on all the mice to cause bone defects. The mice were anesthetized as described above and placed in the supine position. After disinfecting the surgical site, the skin and muscle above the middle shaft of the femur were incised and retracted to expose the bone surface. Then, a precision drill bit was used to create a standardized bone defect (1 mm × 1 mm × 1 mm), which penetrated the cortical bone and extended slightly into the underlying trabecular bone without causing fracture or damaging the opposite cortex on the femur. Subsequently, OVX mice were further randomly divided into three subgroups (*n* = 5 in each group): OVX PBS, OVX CS-βGP and OVX CS-βGP@10Epoxomicin. The SHAM group (*n* = 5) served as another control group. In the SHAM group and the OVX PBS group, the defects were rinsed with PBS but not filled. Conversely, the defects in the OVX CS-βGP and OVX CS-βGP@10Epoxomicin groups were filled with the corresponding hydrogel materials (CS-βGP or CS-βGP@10Epoxomicin). After thorough rinsing, suture the muscle layer and skin layer respectively, and bandage the wound surface. All mice were monitored every day until the end of the experiment. Specimens were collected 3 weeks after bone defect surgery, including the femora, serum and major internal organs.

#### Weight analysis

After modeling, the body weight of mice in each group was measured weekly until the end of the experiment. Based on the recorded weight, draw a weight change curve to evaluate the weight fluctuations during the modeling period.

#### Weight and morphology of the fallopian tube

At 3 weeks post-surgery, fallopian tubes were harvested from mice in each group, gently rinsed with ice-cold PBS and their wet weights were measured and recorded. Morphological assessment was performed by photography to evaluate the shape and size of the tissues.

#### Serum analysis

The mice were deeply anesthetized before the collection of whole blood through cardiac puncture. The blood samples were coagulated at room temperature for 2 h, and then the serum was separated by centrifugation at 3000 rpm for 15 min at 4 °C.

Serum albumin (ALB) levels were quantified using commercial albumin assay kits (Beyotime, Shanghai, China). Simply put, 5 μL of serum was mixed with 200 μL of bromocresol green (BCG) working solution, and the absorbance was measured at 628 nm. The concentration of ALB was determined according to the standard curve.

Blood urea nitrogen (BUN) concentration was determined using urea assay kits (Beyotime, Shanghai, China) in accordance with the manufacturer’s instructions. The serum was mixed with the urea reaction working solution and the absorbance was read at 670 nm. Calculate the BUN concentration using the standard curve of known urea concentration.

Serum alkaline phosphatase (ALP) activity was determined using the alkaline phosphatase assay kit (Beyotime, Shanghai, China). In simple terms, the serum sample was incubated with the chromogenic substrate at 37°C for 5 min and the reaction was terminated with a stop solution. The absorbance was measured at 405 nm, and the ALP activity was determined according to the standard curve.

Serum calcium (Ca^2+^) levels were determined using the calcium colorimetric assay kit (Beyotime, Shanghai, China). Fifty milliliters of serum were mixed with 150 μL of detection working solution, incubated in the dark at room temperature for 10 min and the absorbance was read at 575 nm. The concentration of Ca^2+^ was calculated based on the standard curve.

#### Inflammation of subcutaneous tissue

Following dorsal skin disinfection in 7-week-old mice, 300 μL of PBS, CS-βGP, CS-βGP@5Epoxomicin and CS-βGP@10Epoxomicin were administered via subcutaneous injection. Dorsal tissue was collected 14 days post-injection.

#### Histology staining

Tissue samples, including internal organs, femurs and skin of mice, were fixed in 4% PFA (Coolaber, Beijing, China). After fixation, the femoral specimens were gently stirred in ethylenediaminetetraacetic acid (EDTA, Coolaber, Beijing, China) for 28 days, and the EDTA solution was replaced every 3 days. After the processing was completed, all the samples were embedded in paraffin and sectioned into thin slices 4 μm thick. The rehydrated tissue sections were stained with hematoxylin and eosin (H&E) staining (Beyotime, Shanghai, China). For tartrate resistant acid phosphatase (TRAP) staining, rehydrated tissue sections were incubated with the TRAP working solution (Servebio, Wuhan, China) at 37°C for 30 min. The H&E and TRAP staining images were captured using an upright microscope (OlymPus, Tokyo, Japan).

Immunohistochemical (IHC) staining was performed using TNF-α, IL-1β, p65 (ET1603-12, Hangzhou, Huabio, China), IκBα (1:200, 18220-1-AP, Proteintech, Wuhan, China), Cathepsin K (CTSK, ET1703-44, Hangzhou, Huabio, China) and NFATc1 (ET1704-45, Hangzhou, Huabio, China) antibodies. After antigen recovery, the tissue sections were stained using the Histostein-SP (streptavidin peroxidase) kit (ZSBG-BIO, Beijing, China) in accordance with the manufacturer’s instructions. The staining results were observed under a microscope. Quantitative comparative analysis was conducted by calculating the percentage of positive staining areas using Image J.

#### Micro-CT

Murine femurs were scanned with a micro-CT system (NMC-200, NEMO, Italy). The acquired images were processed for 3D reconstruction using Recon software. Subsequently, bone morphological parameters in the femoral defect region, including bone volume fraction (BV/TV), bone mineral density (BMD), trabecular bone thickness (Tb.Th) and trabecular separation (Tb.Sp), were quantified using Skyscan CTAn software.

#### Data acquisition and differential gene expression analysis

The RNA sequencing dataset GSE198806 was obtained from the Gene Expression Omnibus (GEO) database [25]. This dataset contains a large number of RNA-seq results from bone marrow-derived macrophages in mouse models, including three OVX samples and three SHAM controls. The limma software package was used in R to conduct differential expression analysis between the OVX group and the control group. The threshold for differentially expressed genes (DEGs) was log2FoldChange > 1, and the adjusted *P*-value was <0.05. A group of 200 inflammatory response-related genes (IRRGs) was obtained from the MSigDB database. By calculating the intersection between IRRGs and the identified DEGs, a subset of core target genes that may be involved in inflammation-mediated osteoporosis was obtained. The core target genes were functionally annotated using the clusterProfiler package in R, and gene ontology (GO) enrichment and Kyoto Encyclopedia of Genes and Genomes (KEGG) pathway analysis were conducted. The list of core target genes was submitted to the STRING database to generate the PPI network. The obtained network was imported into Cytoscape for visualization and further topological analysis to identify the key hub genes.

### Statistical analysis

All the above quantitative experiments were repeated at least three times, and the values were expressed as mean ± SD. Statistical analysis was performed using one-way analysis of variance with GraphPad Prism 8.0. *P* < 0.05 is considered significant, and *P* < 0.01 is considered extremely significant.

## Results

### Physicochemical properties of CS-βGP@Epoxomicin

**Figure 2:**
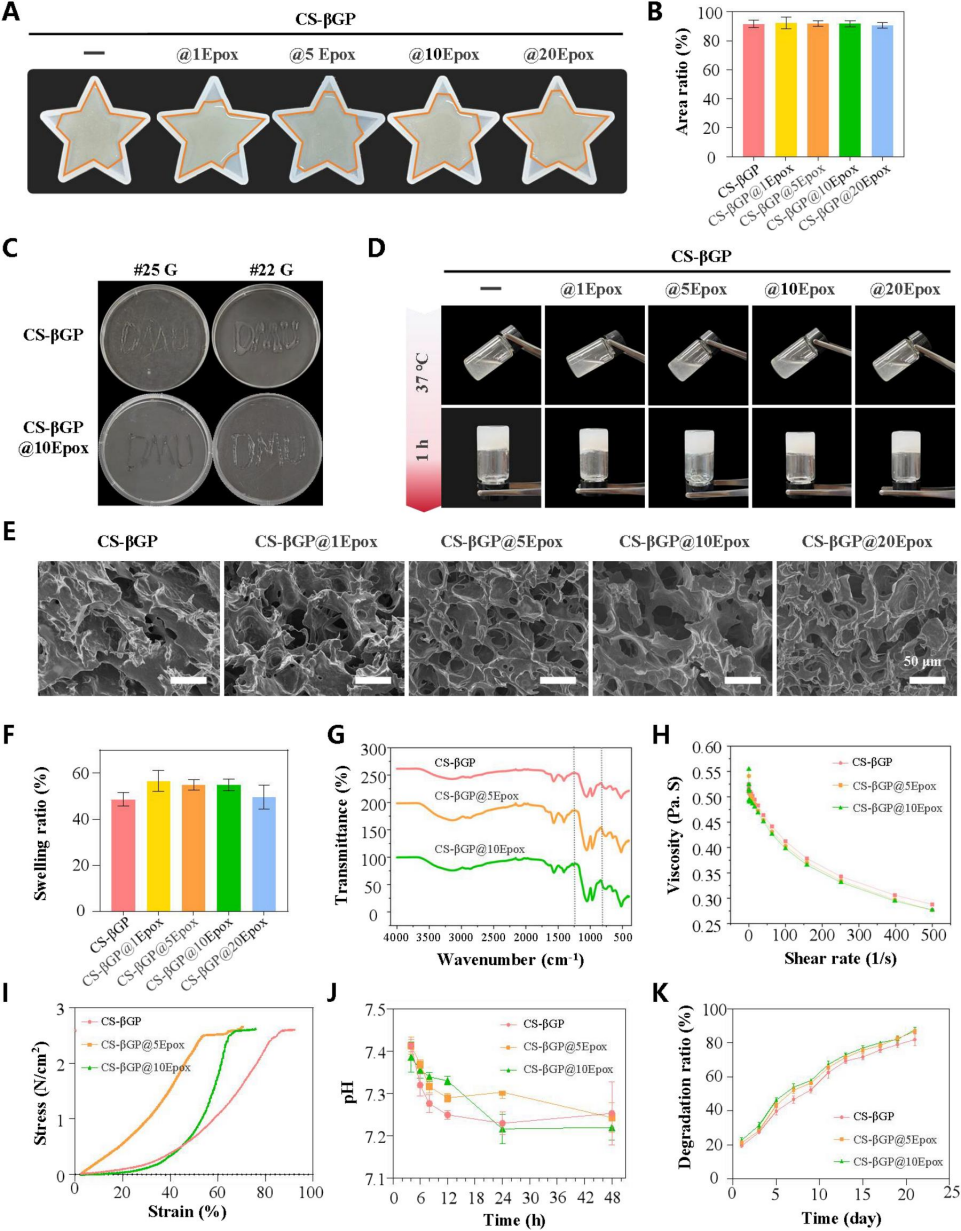
Physicochemical properties of CS-βGP@Epoxomicin hydrogels. (**A**, **B**) Flowability of hydrogels using a pentagram-shaped mold and quantitative analysis of the gelled area percentage. (**C**) Injectability of hydrogels through the 25 G and 22 G needle. (**D**) Gelation time of hydrogels at 37°C. (**E**) SEM images of hydrogels. Scale bar, 50 μm. (**F**) Swelling property of the hydrogels. (**G**) FTIR spectra of hydrogels. (**H**) Shear-thinning behavior of the hydrogels. (**I**) Compressive strength of hydrogels. (**J**) pH of the hydrogels immersed in simulated body fluid over 48 h. (**K**) Degradation property of hydrogels in simulated body fluid during 20 days. CS-βGP, chitosan-β-glycerophosphate disodium hydrogel; Epox, epoxomicin. Data are expressed as mean ± SD. Statistical significance was calculated by one-way ANOVA analysis.

The CS-βGP sodium hydrogel system has good thermal sensitivity, bioadhesion and biocompatibility [26]. Therefore, the composite hydrogel CS-βGP@Epoxomicin with loading epoxomicin was prepared. The modification of CS-βGP through drug loading enhanced the biological functions of the CS-βGP system while maintaining its injectable and *in situ* gelation properties. At room temperature, the hydrogels loaded with different concentrations of epoxomicin exhibited excellent fluidity and successfully filled the star-shaped mold (Figure 2A). Quantitative analysis confirmed that there was no statistically significant difference in flow characteristics among different groups (Figure 2B). Both CS-βGP and CS-βGP@Epoxomicin formulations can be smoothly injected with 22 G and 25 G needles (Figure 2C), indicating that epoxomicin has no effect on the injectability of CS-βGP, making it suitable for skin and bone defects of different sizes. Sol–gel transformation occurred in both CS-βGP and CS-βGP@Epoxomicin 37°C at 1 h of incubation, resulting in stable hydrogels (Figure 2D, Supplementary Figure S1). Chitosan remains in a liquid state under acidic conditions due to electrostatic repulsion. At low temperatures (5–15°C), the addition of β-GP alters the pH value of the system. Glycerol molecules buffer the chitosan amino groups, forming a hydrated layer that maintains solubility at neutral pH. When heated to physiological temperature, the hydrated layer weakens, enhancing hydrophobic interactions and hydrogen bonds, thereby driving the sol–gel transition [27]. Since epoxomicin has no obvious chemical interaction with CS or βGP and is encapsulated in the hydrophobic region of the hydrogel network, its incorporation does not significantly alter the gelation time or temperature of the system. Swelling tests indicated that the swelling rates of CS-βGP and CS-βGP@Epoxomicin hydrogels were between 40% and 60% (Figure 2F), belonging to low-swelling hydrogels (typically 0–150%) [28]. This low-swelling property is conducive to maintaining the structural integrity and mechanical stability of the hydrogel *in vivo* and preventing potential erosion of the surrounding bone tissue due to excessive swelling. In addition, the relatively dense gel network is conducive to effective drug encapsulation and the sustained release of epoxomicin. SEM imaging revealed that both CS-βGP and CS-βGP@Epoxomicin hydrogels had 3D, porous and uniformly distributed network structures (Figure 2E). This microstructure provides space for cell attachment and migration as well as channels for the transportation of nutrients [29]. It also enhances the drug delivery efficiency, prevents the complete release of the drug within a short period of time and thereby promotes the long-term local biological activity of epoxomicin. The physicochemical properties and microstructure of CS-βGP@Epoxomicin identified that the addition of different concentrations of epoxomicin remains the basic characteristics of the hydrogel. Based on these findings, CS-βGP, CS-βGP@5Epoxomicin and CS-βGP@10Epoxomicin were selected for subsequent performance evaluation.

FTIR analysis further indicated that there were identical characteristic peaks between the CS-βGP and CS-βGP@Epoxomicin hydrogels, with no detectable new chemical bonds (Figure 2G). Given that the gelation temperature and time of the system remained unchanged, it can be inferred that epoxomicin was primarily physically embedded within the hydrophobic micro-regions of the hydrogel network.

Rheological measurements further evaluated the shear-thinning performance of the injectable hydrogel. As shown in Figure 2H, the apparent viscosities of CS-βGP, CS-βGP@5Epoxomicin and CS-βGP@10Epoxomicin decreased significantly with the increase of shear rate. This inverse relationship between viscosity and shear rate is a decisive characteristic of non-Newtonian shear-thinning fluids [30]. The experimental results verified that the CS-βGP@Epoxomicin hydrogels retained the basic shear-thinning and thixotropic properties of the CS-βGP-based material. The compressive mechanical properties characterized the structural integrity of CS-βGP and CS-βGP@Epoxomicin hydrogels and their applicability to bone defect applications. The representative stress–strain curves are shown in Figure 2I. All hydrogel groups exhibited the compression behavior characteristic of elastoplastic porous materials. At low strain (<15%), it was observed that the slopes of CS-βGP and CS-βGP@10Epoxomicin in the initial linear elastic region were similar, confirming that the incorporation of epoxomicin maintains compressive modulus of the hydrogel matrix. After linear deformation, CS-βGP@10Epoxomicin yielded at approximately 40% strain, significantly earlier than the CS-βGP. This earlier yield point indicates a moderate increase in the structural brittleness of the gel loaded with chemicals [31]. Subsequently, a wide stress plateau area (approximately 60–100% strain) was detected, corresponding to the progressive collapse of the porous network. The results showed that although the epoxomicin loading maintained the initial stiffness (compressive modulus) of the CS-βGP hydrogels, it slightly advanced its yield point. This phenomenon might be due to the introduction of microscopic heterogeneity by hydrophobic drug molecules in the polymer network. This phenomenon is likely attributable to the introduction of microscopic heterogeneities within the polymer network by the hydrophobic drug molecules.

The pH stability and degradation behavior of CS-βGP@Epoxomicin were further assessed using SBF *in vitro*. As shown in Figure 2J, the pH of the soaking medium remained within the physiological range of 7.2–7.4 for the first 48 h, indicating that no acidic or alkaline by-products were released during the initial dissolution of hydrogels. Meanwhile, hydrogels displayed progressive degradation characteristics (Figure 2K), with a mass loss of approximately 40% after 5 days and a degradation of over 80% of the hydrogel matrix within 20 days. The pH stability and degradation rate remained consistent among the three groups. The results of rheological, compressive mechanics, chemical structure and degradation analyses *in vitro* demonstrated that epoxomicin was mainly physically encapsulated in the CS-βGP hydrogel matrix without forming significant chemical cross-links with the polymer network. Therefore, the incorporation of epoxomicin did not alter the fundamental physicochemical or mechanical properties of the hydrogel.

### The biological effects of CS-βGP@epoxomicin *in vitro*

**Figure 3:**
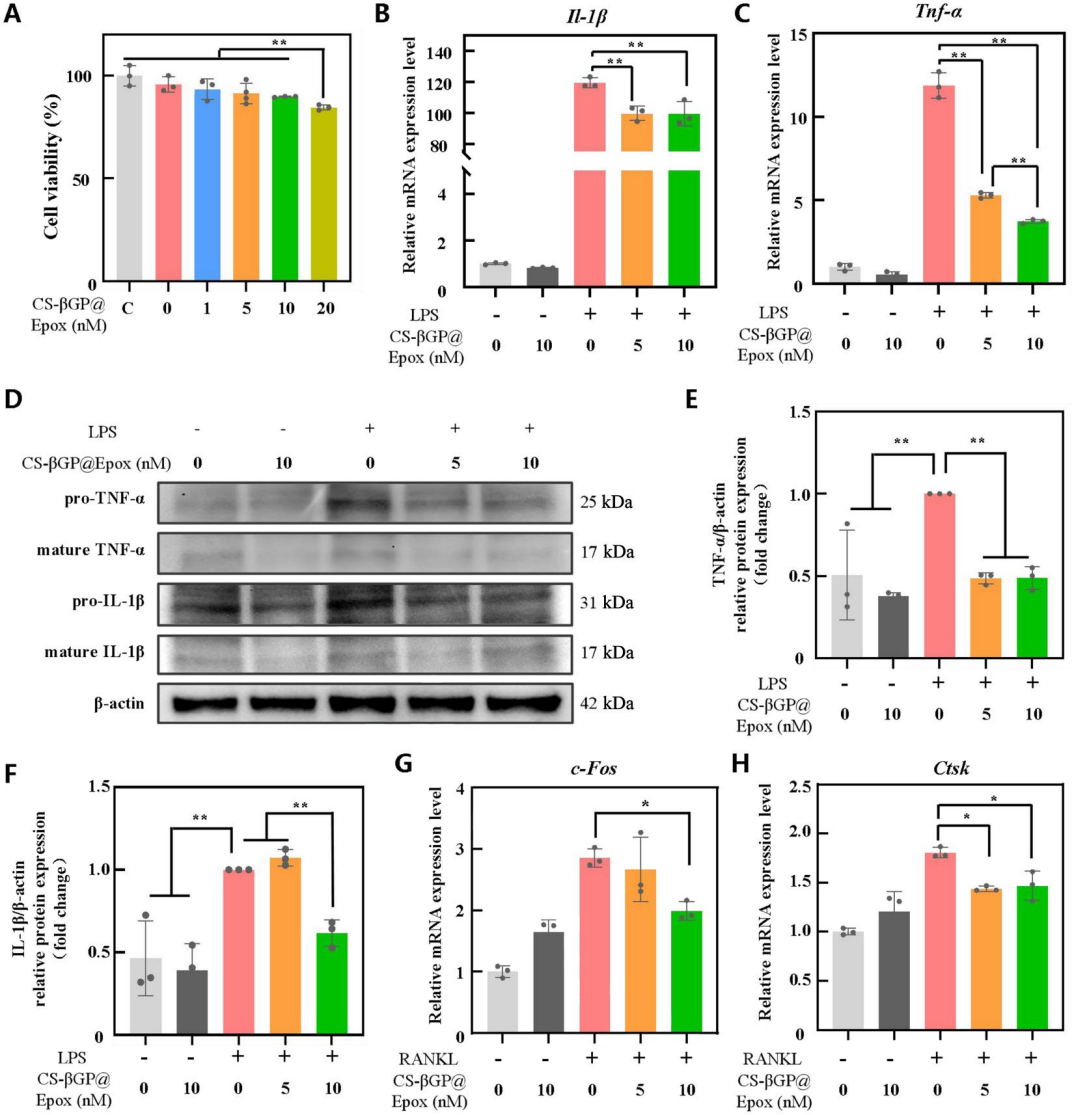
Inflammatory response and osteoclast differentiation effects of CS-βGP@Epoxomicin hydrogels *in vitro*. (**A**) Viability of BMMs treated with CS-βGP hydrogel extract containing different concentrations of Epoxomicin, as assessed by CCK-8 assay. (**B, C**) Relative mRNA expression levels of the pro-inflammatory cytokines *Il-1β* and *Tnf-α* in BMMs treated with LPS and hydrogel extract for 6 h. (**D**) Protein expression levels of IL-1β and TNF-α in BMMs treated with LPS and hydrogel extract for 6 h. (**E, F**) Quantitative analysis of the Western blot results. (**G, H**) Relative mRNA expression levels of osteoclast-related genes *c-fos* and *Ctsk* in BMMs treated with RANKL and hydrogel extract for 3 days. C, control; CS-βGP, chitosan-β-glycerophosphate disodium hydrogel; Epox, epoxomicin. Data are expressed as mean ± SD. Statistical significance was calculated by one-way ANOVA analysis. *P < 0.05, **P < 0.01.

The physicochemical properties of epoxomicin-loaded hydrogels are beneficial for the growth and repair of cells and tissues. Therefore, this study evaluated the biological effects of CS-βGP and CS-βGP@10Epoxomicin hydrogels through experiments *in vitro*. The CCK-8 assay showed that cell viability gradually decreased with the increase of drug loading concentration, among which the cell viability of the CS-βGP@20Epoxomicin group decreased significantly (Figure 3A). This indicated that a higher loading concentration enhanced the functional effect of drug release within the drug concentration range of 0–20 nM, but a drug loading concentration higher than 20 nM affected the biocompatibility of the hydrogel. Based on their biological safety and efficacy, CS-βGP@5Epoxomicin and CS-βGP@10Epoxomicin were selected for subsequent experiments.

BMMs play a key role in bone repair by participating in immune responses and bone remodeling [32]. Mature BMMs release pro-inflammatory cytokines under LPS stimulation. The RT-qPCR results showed that CS-βGP@5Epoxomicin and CS-βGP@10Epoxomicin significantly downregulated the expressions level of *Il-1β* and *Tnf-α* under LPS stimulation (Figure 3B and C). Western blot analysis further revealed that CS-βGP@5Epoxomicin downregulated TNF-α protein only, while CS-βGP@10Epoxomicin downregulated the expression of IL-1β and TNF-α proteins (Figure 3D–F). These findings confirmed that CS-βGP@10Epoxomicin effectively mitigates the inflammatory response of BMMs at the transcriptional and translational levels. The immunofluorescence analysis demonstrated that LPS stimulation induced pronounced nuclear translocation of p65, accompanied by a marked reduction in IκBα protein expression. In contrast, treatment with CS-βGP@10Epoxomicin or NF-κB inhibitor effectively preserved IκBα levels and retained p65 in the cytoplasm, indicating that epoxomicin inhibits NF-κB activation primarily by preventing IκBα degradation (Supplementary Figure S2).

In addition, BMMs differentiate into osteoclast under RANKL stimulation, which can disrupt bone remodeling balance. RT‑qPCR results displayed that CS-βGP@5Epoxomicin downregulated only *Ctsk* expression, while CS-βGP@10Epoxomicin significantly reduced the expression of both *c‑Fos* and *Ctsk* (Figure 3G and H). Taken together, the cellular experiments demonstrated that CS-βGP@10Epoxomicin remains within a biosafe concentration range and effectively mitigates inflammation while inhibiting osteoclast differentiation.

### Evaluation of the biological characteristics of CS-βGP@Epoxomicin *in vivo*

CS-βGP@10Epoxomicin has a significant ability to inhibit inflammation and osteoclast differentiation *in vitro*. This study further evaluated its biosafety, anti-inflammatory efficacy and ability to maintain bone remodeling balance in an animal model of osteoporosis *in vivo*, providing a theoretical basis for the future clinical transformation and application of this hydrogel.

#### Construction of osteoporosis model

**Figure 4:**
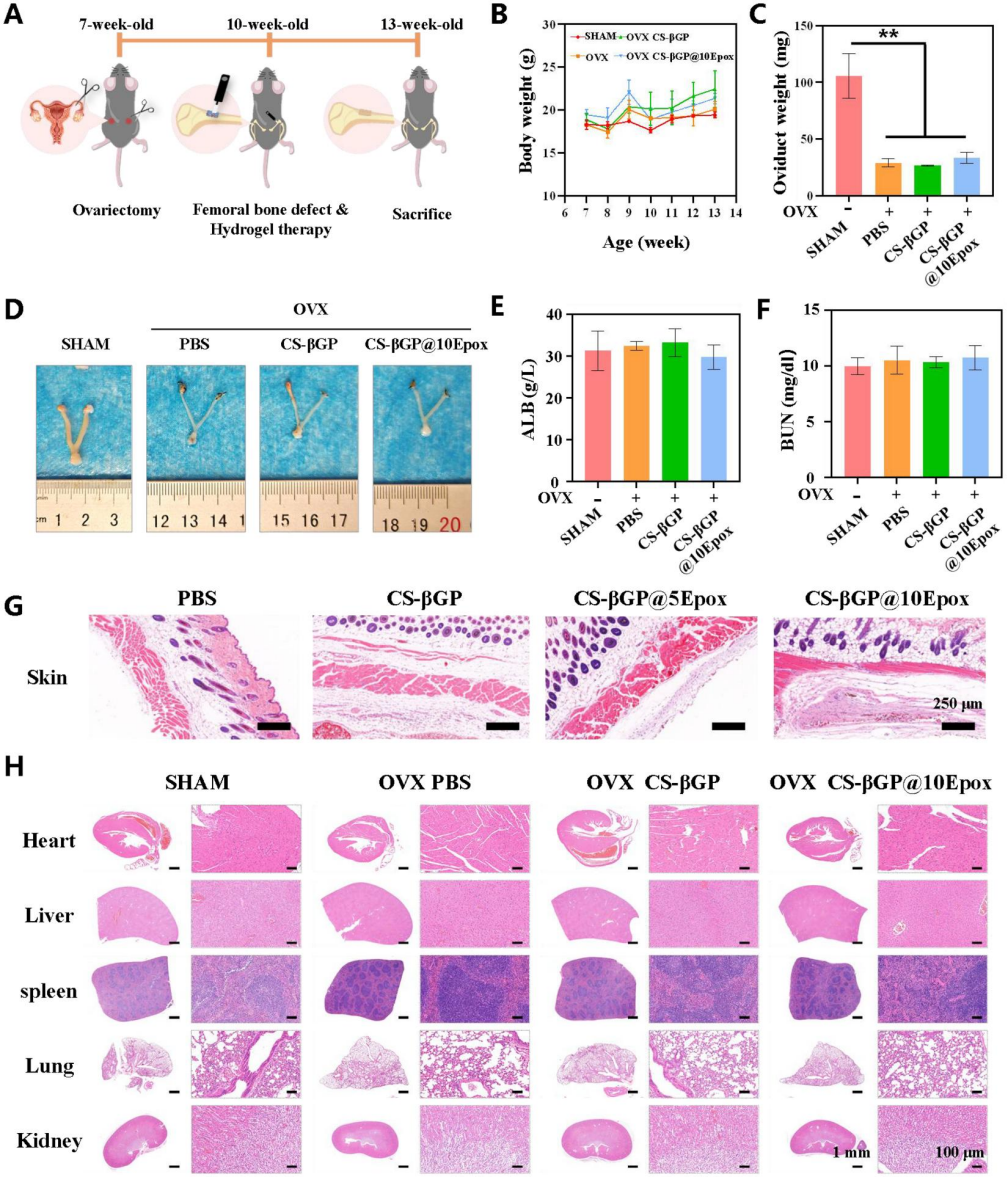
Biological safety evaluation of CS-βGP@Epoxomicin hydrogel *in vivo*. (**A**) Schematic illustration of the surgical procedure for animal model establishment and the subsequent tissue collection schedule. (**B**) Body weight changes of mice after hydrogel treatment. (**C**) Weight of the fallopian tubes from hydrogel-treated mice. (**D**) Morphology of the fallopian tubes from hydrogel-treated mice. (**E, F**) ALB and BUN concentrations in serum of mice. (**G**) H&E staining of major organs and local skin tissue following subcutaneous injection, and assessment of the inflammatory reaction induced by the subcutaneously injected hydrogel. (**H**) H&E staining images of the heart, liver, spleen, lung and kidney from mice. Scale bars, 1 mm (main image) and 100 μm (enlarged view). CS-βGP, chitosan-β-glycerophosphate disodium hydrogel; Epox, epoxomicin; OVX, ovariectomy. Data are expressed as mean ± SD. Statistical significance was calculated by one-way ANOVA analysis. **P < 0.01.

The OVX mouse model is a mature animal model that simulates postmenopausal osteoporosis. Therefore, this study established an OVX model to investigate bone defect repair under osteoporosis conditions (Figure 4A). The results of the body weight analysis showed that in the first week after ovariectomy, the body weight of all mice briefly decreased and then recovered in the second week. During this period, the body weight of OVX mice was always higher than that of the SHAM group. After the bone defect surgery in the third week, the weight of mice dropped briefly again. The body weight of the mice steadily recovered from the fourth to the sixth week, and the body weight of OVX mice was higher than that of SHAM mice (Figure 4B). Uterine mass analysis displayed that the uterine mass of OVX mice was significantly reduced compared with the SHAM group (Figure 4C). Morphological examination of the uterus further confirmed that compared with SHAM mice, the uterus of OVX mice was significantly atrophied and its size was reduced (Figure 4D). Overall, the weight change pattern, as well as the assessment of uterine morphology and weight, verified the successful establishment of the OVX model.

#### Biocompatibility evaluation of CS-βGP@Epoxomicin *in vitro*

CS-GP is a biodegradable thermosensitive hydrogel [19]. Its degradation products (such as oligosaccharides, glucosamine, glycerol and phosphate ions) are all natural products and have good biocompatibility [15, 33]. However, epoxomicin is a proteasome inhibitor, and its biocompatibility needs further assessment. In this study, the biocompatibility of the material was systematically examined from the local to the systemic level, including local inflammatory responses at the implantation site, changes in systemic serum biomarkers and histopathological alterations in distant major organs.

The results of subcutaneous local injection exhibited that there was no obvious inflammatory cell infiltration in the skin tissue at the implantation site of CS-βGP or CS-βGP@10Epoxomicin compared with normal tissue (Figure 4G). There were no significant differences in ALB and BUN levels among the groups (Figure 4E and F). No obvious lesions in the heart, liver, spleen, kidneys or lungs were observed in CS-βGP and CS-βGP@10Epoxomicin (Figure 4H). In conclusion, these results confirmed that CS-βGP@10Epoxomicin is a safe, reliable, biodegradable and renewable material.

#### The role of CS-βGP@Epoxomicin in bone defect repair

**Figure 5:**
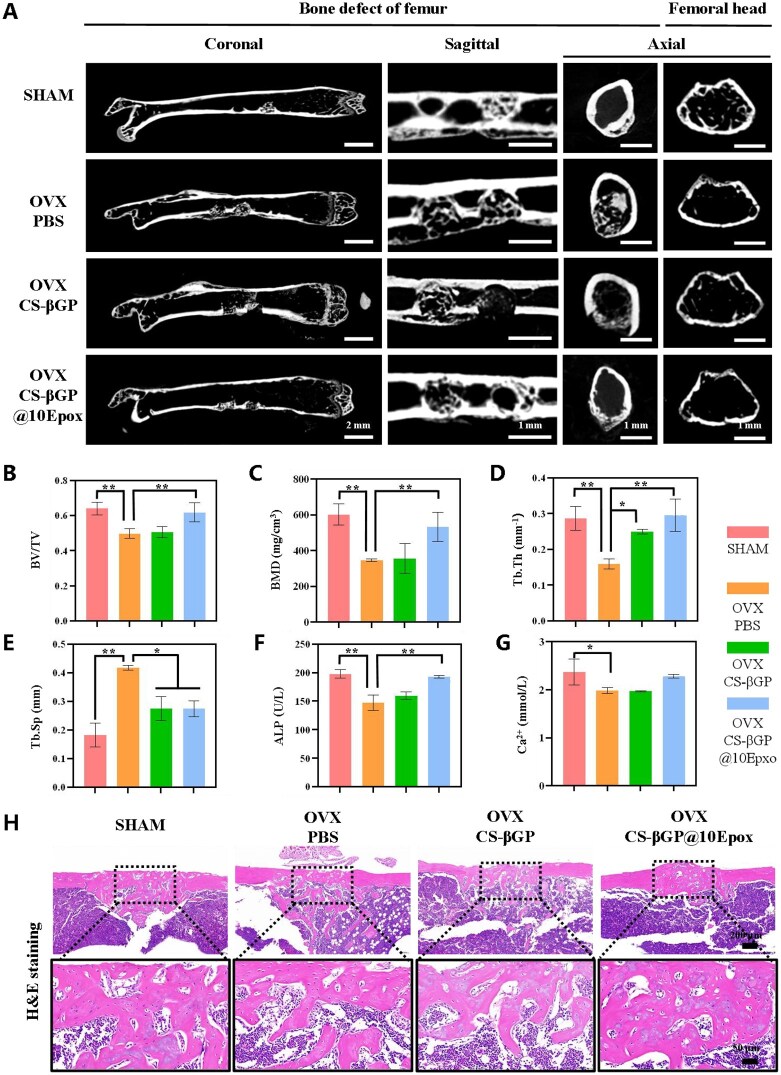
Evaluation of bone loss of OVX mice treated with CS-βGP@Epoxomicin hydrogel *in vivo*. (**A**) Representative μ-CT images of the bone defect area. Scale bars, 2 mm (coronal images) and 1 mm (axial and sagittal images). (**B–E**) Quantitative micro-CT analysis of the bone defect region: (**B**) BV/TV, (**C**) BMD, (**D**) trabecular number (Tb.N) and (**E**) Tb.Sp. (**F, G**) ALP and Ca^2+^ concentration in serum of mice. (**H**) Histological images of the bone defect surrounding area stained with H&E. Dashed lines indicate the original bone defect region. Scale bars, 200 μm (main image) and 50 μm (enlarged view). CS-βGP, chitosan-β-glycerophosphate disodium hydrogel; Epox, epoxomicin; OVX, ovariectomy. Data are expressed as mean ± SD. Statistical significance was calculated by one-way ANOVA analysis. *P < 0.05, **P < 0.01.

The bone remodeling balance of patients with osteoporosis is disrupted, leading to a decrease in bone healing ability [34]. Therefore, the osteoporosis model was adopted to further evaluate the role of CS-βGP@10Epoxomicin in the process of bone repair. The μCT results showed that the trabeculae of the femur were thinly broken, the area of bone defect increased, the cortical bone plate connection was incomplete and the trabeculae were thin and unorganized in OVX mice. The bone repair effect in the CS-βGP@5Epoxomicin group was similar to that in the OVX PBS group. In the CS-βGP@10Epoxomicin group, the area of bone defect was significantly reduced, the cortical bone plates were completely connected, the trabeculae became thicker and the arrangement was denser (Figure 5A). μCT analysis revealed that BV/TV, BMD, Tb.Th were significantly decreased in the OVX PBS group, while these parameters were significantly improved in the CS-βGP@10Epoxomicin group (Figure 5B–D). Furthermore, Tb.Sp significantly increased in the OVX PBS group, and this parameter significantly improved in the CS-βGP@10Epoxomicin group (Figure 5E). Serum ALP concentration increased in the CS-βGP@10Epoxomicin treatment group (Figure 5F). The serum calcium concentration in the OVX PBS group was significantly lower than that in the SHAM group. Although the CS-βGP@10Epoxomicin group also exhibited decreased serum calcium levels, there was no significant difference compared with the OVX PBS group (Figure 5G). The H&E staining results depicted that the new bone tissue in the OVX PBS group and the CS-βGP@5Epoxomicin group was loose and dispersed, while the mature bone tissue was relatively scarce. In contrast, at the bone defect sites treated with CS-βGP@10Epoxomicin, the new trabeculae were regularly arranged, structurally dense and contained more mature bone tissue (Figure 5H). These results demonstrated that CS-βGP@10Epoxomicin inhibited bone loss of osteoporotic bone defects.

**Figure 6:**
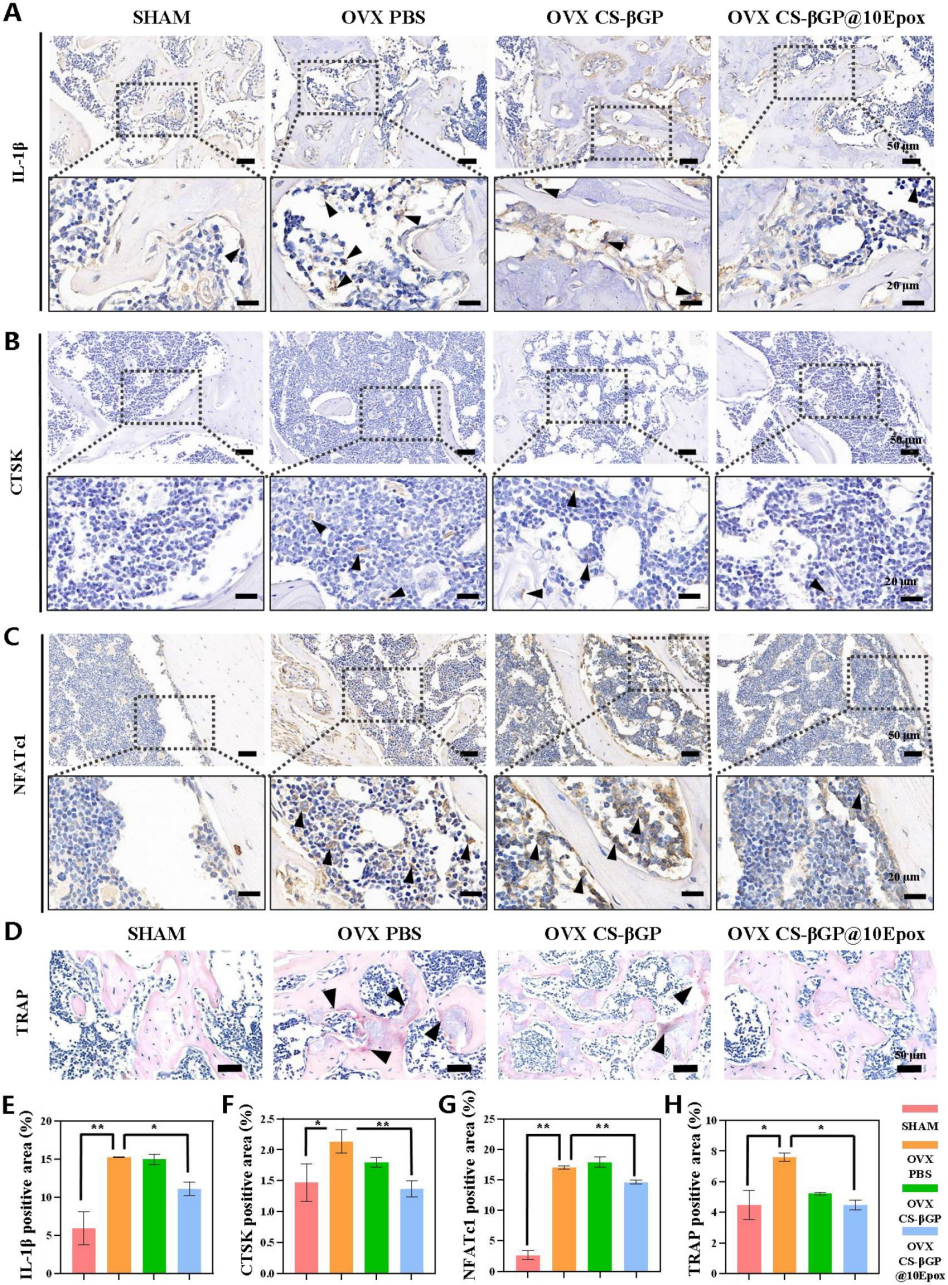
Evaluation of inflammatory and osteoclast differentiation of OVX mice treated with CS-βGP@Epoxomicin hydrogel *in vivo*. (**A–C**) IHC staining of the bone defect area for the pro-inflammatory and osteoclast differentiation-related proteins IL-1β, CTSK and NFATc1. Scale bars, 50 μm (main image) and 20 μm (enlarged view). (**D**) TRAP-positive osteoclast staining images of the bone defect surrounding area. Scale bar, 50 μm. (**E–H**) Quantitative analysis of IL-1β, CTSK and NFATc1 and TRAP-positive cells from staining images. CS-βGP, chitosan-β-glycerophosphate disodium hydrogel; Epox, epoxomicin; OVX, ovariectomy. Data are expressed as mean ± SD. Statistical significance was calculated by one-way ANOVA analysis. *P < 0.05, **P < 0.01.

The main pathogenesis of osteoporosis includes excessive bone resorption and intensified inflammatory response [35]. Therefore, IHC staining was used to further investigate the mechanism by which CS-βGP@10Epoxomicin inhibits bone loss through dual anti-inflammatory and anti-osteoclast differentiation effects. On the one hand, the proportion of cells positive for the inflammation-related protein IL-1β in the OVX PBS group increased significantly, confirming the elevated inflammatory activity near the bone defect site in OVX mice. CS-βGP@10Epoxomicin treatment reduced the proportion of IL-1β positive cells (Figure 6A and E), invalidating that it could inhibit the inflammatory response around bone defects in OVX mice. On the other hand, the expression rates of osteoclast differentiation-related proteins CTSK and NFATc1 in OVX mice were significantly increased, identifying that osteoclasts were actively differentiated in the bone tissue around the defect. After CS-βGP@10Epoxomicin treatment, the proportion of CTSK and NFATc1 positive cells decreased (Figure 6B, C, F and G), proving that CS-βGP@10Epoxomicin inhibited osteoclast differentiation around bone defects in OVX mice. TRAP staining showed results consistent with the IHC findings (Figure 6D and H). In conclusion, the results of these *in vivo* experiments indicated that CS-βGP@10Epoxomicin restored bone repair and regeneration capabilities by weakening local inflammatory responses and osteoclast differentiation at bone defect sites.

### Analysis of genes related to inflammatory responses in osteoporosis

**Figure 7:**
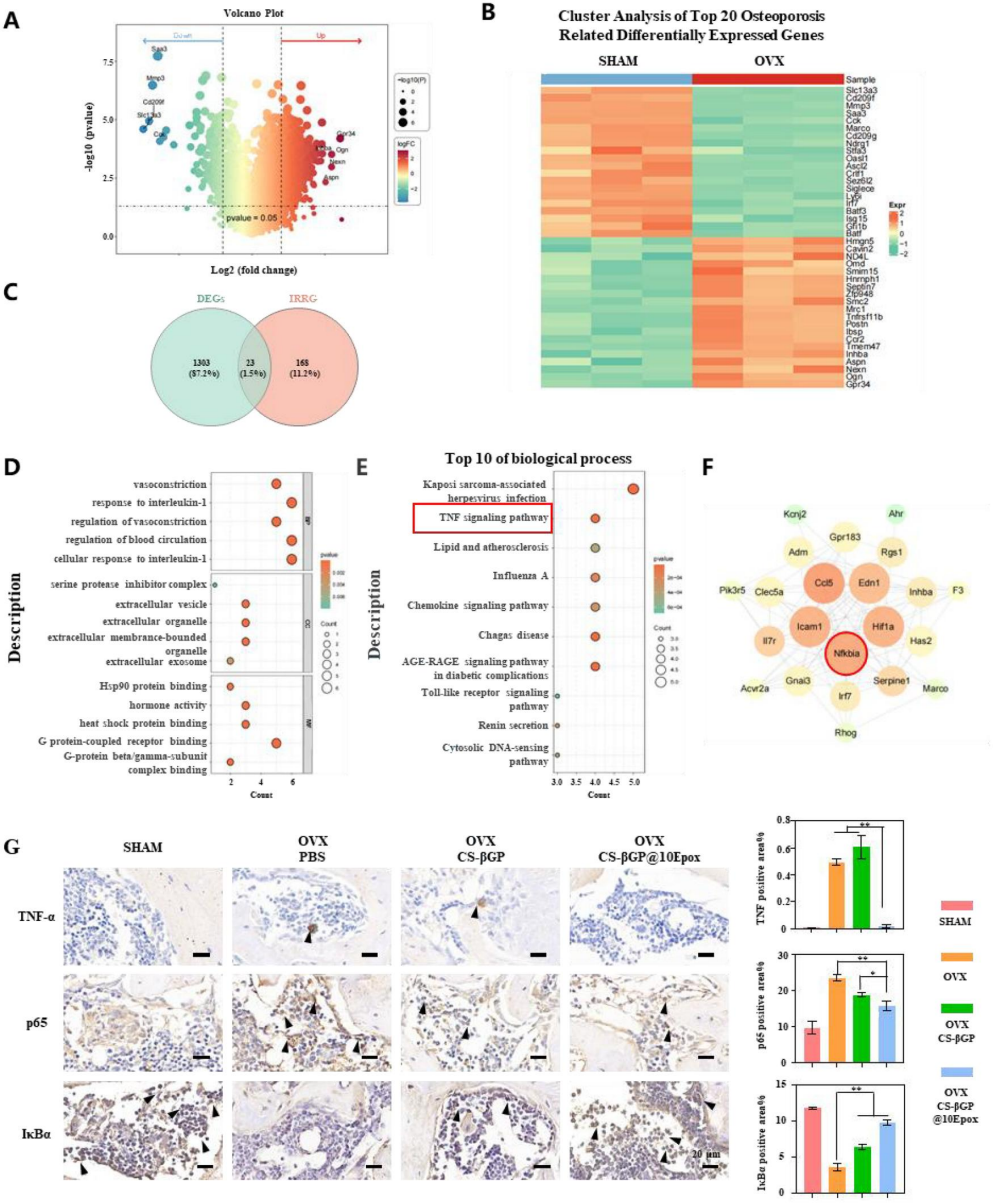
Transcriptome sequencing analysis of the anti-inflammatory mechanisms in osteoporosis. (**A**) Volcano plot of DEGs between normal and osteoporosis patients. Dots represent up- and down-regulated genes, respectively. (**B**) Heatmap of top 20 DEGs between normal and osteoporosis patients. Different colors indicate up- and down-regulation, respectively. (**C**) Venn diagram analysis of osteoporosis-related DEGs and IRRGs. (**D, E**) GO and KEGG enrichment analyses of biological pathways and mechanisms associated with osteoporosis. (**F**) PPI network of inflammatory-osteoporosis genes constructed from the STRING database. (**G**) IHC staining and quantitative analysis of TNF-α, p65 and IκBα. Scale bar, 20 μm. CS-βGP, chitosan-β-glycerophosphate disodium hydrogel; Epox, epoxomicin; OVX, ovariectomy. Data are expressed as mean ± SD. Statistical significance was calculated by one-way ANOVA analysis. *P < 0.05, **P < 0.01.

To validate the pathological features of the OVX model and investigate the inflammatory mechanisms in osteoporosis, we performed bioinformatics mining on a public bulk RNA-seq dataset (GSE198806) of mouse bone marrow-derived macrophages. Differential expression analysis between the OVX and SHAM groups identified 1326 genes meeting the criteria of |log2 fold change| > 1 and an adjusted *P*-value <0.05, which were defined as osteoporosis-related DEGs. A volcano plot illustrated the overall distribution of these DEGs, comprising 1134 upregulated and 192 downregulated genes (Figure 7A). Hierarchical clustering of the expression patterns of the top 20 DEGs is presented in a heatmap (Figure 7B). From the MSigDB database, a curated set of 200 IRRGs was obtained. The intersection of these IRRGs with the osteoporosis-related DEGs identified 23 core inflammatory genes potentially central to osteoporosis (Figure 7C). The molecular functions and pathways of these 23 core genes were clarified through GO and KEGG enrichment analyses. The analysis of GO includes three categories: biological processes (BP), cellular components and molecular functions (MF) [5]. The core inflammatory genes were mainly enriched in BP, with 990 significant items. The top five differentially enriched BP terms included vasoconstriction, vascular process regulation, blood circulation and IL-1 related reaction processes (Figure 7D). KEGG pathway annotations revealed the top 10 pathways with the most significant enrichment, among which the TNF signaling pathway was the most prominent inflammation-related pathway (Figure 7E). In addition, a PPI network was constructed for the proteins encoded by these 23 core genes. Network topology analysis identified several key hub proteins, including Nfkbia, Ccl5, Icam1, Hif1a and Edn1 (Figure 7F). These hub proteins, characterized by high connectivity within the network, were regarded as potential key molecular targets for regulating the inflammatory response in osteoporosis.

IHC analysis was performed on the bone defect area to verify the activation of inflammatory pathways predicted by transcriptome analysis and evaluate the therapeutic effect of hydrogels. Compared with the SHAM group, the positive signals of p65 protein and TNF-α in the cells around the bone defect area (mainly the bone marrow cavity and endothelium) in the OVX PBS group were significantly enhanced (Figure 7G). Nuclear translocation of p65 is a direct morphological indicator of the activation of the NF-κB transcriptional complex [36]. The positive results of p65 protein and TNF-α confirmed the continuous activation of the TNF-NF-κB inflammatory signaling pathway in the local bone microenvironment under osteoporosis, which was consistent with the pathway enrichment results of the bioinformatics analysis of DEGs. Furthermore, IHC analysis revealed that, compared with the SHAM group, positive staining for IκBα in cells surrounding the bone defect area was markedly weakened in the OVX PBS group, corresponding to the pathological feature of continuous IκBα phosphorylation and degradation that drive persistent NF-κB pathway activation in the osteoporotic microenvironment [37]. Importantly, CS-βGP@10Epoxomicin treatment effectively reversed these pathological changes. In the bone tissue of mice treated with CS-βGP@10Epoxomicin, the nuclear localization of p65 and the expression level of TNF-α were significantly decreased, while the expression level of IκBα was significantly increased (Figure 7G). Further quantitative analysis showed the statistically significant difference between the two groups. These results suggested that CS-βGP@10Epoxomicin could effectively inhibit the key TNF-NF-κB inflammatory signaling axis in local osteoporotic bone defects.

## Discussion

This study successfully constructed and validated the efficacy and safety of thermosensitive hydrogel (CS-βGP@Epoxomicin) for the treatment of osteoporotic bone defects. The system not only has favorable physicochemical properties and biocompatibility, but also promotes bone repair by inhibiting the NF-κB signaling pathway to suppress inflammation and regulate the differentiation of osteoclasts.

The CS-βGP thermosensitive hydrogel system constructed in this study demonstrates core advantages, including injectability, body-temperature-responsive *in situ* gelation capability and favorable biocompatibility. Experimental results confirmed that even after drug loading, the system maintained good fluidity and shear-thinning behavior, facilitating minimally invasive implantation via syringe (Figure 2A–C and H). It also exhibited a uniform and interconnected porous microstructure, which is conducive to cell growth and drug release, while providing a certain degree of mechanical strength to offer space for bone tissue growth (Figure 2E) [38]. FTIR analysis of the drug-loaded hydrogel showed no generation of new chemical bond signals (Figure 2G). Combined with the observation that the gelation temperature and time remained essentially unchanged, it was inferred that the drug was primarily dispersed within the hydrogel network through physical encapsulation [39]. This physical loading mechanism maximally preserves the original chemical structure and cross-linked network integrity of the hydrogel, effectively avoiding issues such as drug burst release or incomplete release that may be caused by chemical bonding. It ensures the bioactivity of the drug while avoiding potential structural uncertainties or toxicity associated with chemical modifications. This approach is similar to recently reported thiolated chitosan composite hydrogel systems that achieve drug loading through physical encapsulation [40]. In contrast to some bone repair materials that rely on complex chemical cross-linking or incorporation of inorganic components (e.g. hydroxyapatite) to enhance mechanical strength, the design focus of this system lies in retaining the thermosensitive hydrogel properties while introducing active drugs (such as epoxomicin) via physical encapsulation. This strategy aims to regulate the osteoporotic bone microenvironment while providing a certain degree of mechanical support and a platform for localized drug release for bone repair.

In terms of biological function, CS-βGP@10Epoxomicin hydrogel demonstrates a powerful ‘dual-regulatory’ capability. *In vitro* experiments confirmed that this system effectively reduces the expression of the pro-inflammatory cytokines IL-1β and TNF-α in LPS-stimulated BMMs (Figure 3B–F), and inhibits the expression of key genes (*c-Fos*, *Ctsk*) involved in the differentiation of osteoclasts induced by RANKL (Figure 3G–H). These results are in line with trends in treatment strategies for the complex pathological microenvironment of osteoporotic bone defects. For example, one study built a dual-device self-healing hydrogel containing benzyl 3β-amino-11-oxo-olean-12-en-30-oate (GN-Bn) that promotes the healing of MRSA-infected wounds through synergistic antibacterial and anti-inflammatory effects [41]. Similarly, our strategy aims to simultaneously intervene in two major pathological processes that coexist in osteoporotic bone defects: ‘excessive inflammation’ and ‘abnormal bone resorption’. Epoxomicin blocks nuclear translocation and activation of NF-κB by inhibiting IκBα degradation, thereby suppressing the release of downstream inflammatory factors and the initiation of osteoclast differentiation at the source [9]. In our immunofluorescence results, the restoration of IκBα expression by CS-βGP@10Epoxomicin confirmed this (Supplementary Figure S2). This ‘upstream targeting, dual inhibition’ mechanism can offer more fundamental and broader regulatory effects compared to exclusively inhibiting downstream molecules (e.g. a single inflammatory cytokine or protein).

*In vivo* experiments have verified that the system provides good biocompatibility (Figure 4), while further confirming its therapeutic potential. μCT and histological analyses showed that CS-βGP@10Epoxomicin treatment significantly improved bone microstructure parameters (e.g. increased BV/TV, BMD, Tb.Th and decreased Tb.Sp) and promoted the formation of more regular and dense new bone (Figure 5). These results are highly similar to the SHAM group. IHC results further elucidated its mechanism, showing that the treatment significantly reduced the proportion of IL-1β-positive cells at the local bone defect site and inhibited the expression of the osteoclast marker proteins TRAP, CTSK and NFATc1 (Figure 6). This clearly indicates that the hydrogel system, through the local sustained release of epoxomicin, effectively remodels the bone repair microenvironment, shifting the pro-inflammatory, pro-osteoclast imbalance toward a pro-repair direction. This local targeted delivery strategy cleverly circumvents potential side effects such as neurotoxicity associated with systemic administration of epoxomicin [42], aligning with the design philosophy of novel drug delivery systems aimed at prolonging local drug action time and improving bioavailability.

Finally, this study utilized bioinformatics mining of public data to identify the activation of inflammatory signaling pathways under osteoporotic conditions. Subsequently, histological validation using an OVX mouse bone defect model demonstrated that the CS-βGP@10Epoxomicin hydrogel can exert its therapeutic effects through local intervention via the NF-κB pathway (Figure 7). Bioinformatics analysis showed that DEGs in BMMs of OVX mice intersected with inflammation-related genes, and 23 core genes were screened (Figure 7A–C). In the PPI network analysis, the key protein IκBα in the NF-κB pathway showed high connectivity (Figure 7F). IκBα is the core inhibitory protein in the NF-κB signaling pathway and maintains NF-κB in an inactive state in the cytoplasm by binding to and masking its nuclear localization signal [43]. Furthermore, our IHC analysis showed that IκBα expression was significantly upregulated in the bone tissue of mice treated with CS-βGP@10Epoxomicin (Figure 7G). These results further demonstrated that CS-βGP@10Epoxomicin modulates the key NF-κB signaling pathway in osteoporotic bone defects by targeting IκBα. KEGG analysis showed significant enrichment of the TNF signaling pathway, which represents an inflammation pathway (Figure 7E). This result is highly consistent with previous research showing that the NF-κB signaling pathway, as a center that regulates the differentiation of the osteoclasts and inflammatory responses, plays a crucial role in the pathological progression of osteoporosis [44, 45]. For instance, it has been confirmed that plastrum testudinis extract treats senile osteoporosis through the NF-κB signaling pathway [46]. Another earlier study also pointed out that in the bone tissue of osteoporotic model mice, the expression of NF-κB subunits (such as p50, p65) within osteoclasts and their downstream pro-inflammatory factors (such as IL-6, IL-1β, TNF-α) was significantly upregulated [47]. Our data corroborate these findings, collectively revealing the central role of the TNF-α and NF-κB signaling pathways in driving osteoporosis-related inflammation and bone resorption. More importantly, our immunohistochemistry results provided direct evidence at the tissue level: In the OVX mouse bone defect area, the accumulation of protein p65 (a key nuclear translocation protein of the NF-κB pathway) in cell nuclei and expression of TNF-α were significantly increased, which visually confirmed the transcriptional activation of the NF-κB pathway and the existence of an inflammatory microenvironment. Following treatment with the CS‑βGP@10Epoxomicin hydrogel, the expression of both p65 and TNF-α was significantly inhibited, while IκBα expression was elevated (Figure 7G). This clearly demonstrates that this delivery system effectively interferes with the NF-κB signaling pathway through local persistent release of epoxomicin, thereby reversing the pathological condition of osteoporotic bone repair.

This study still has the following limitations: it lacks systematic tracking and evaluation of the long-term, complete bone remodeling process (including remodeling and maturation) and validation in large animal models or more complex defect scenarios is absent. Secondly, constrained by the precision of current *in vitro* drug release detection technologies, the precise sustained-release kinetics of the highly potent loaded drug (such as epoxomicin) cannot be clearly quantified. Furthermore, the strategy primarily focuses on inhibiting osteoclast activity and inflammation; loading other drugs or growth factors (such as BMP-2) to achieve synergistic ‘anti-inflammatory + pro-osteogenic’ effects and develop the platform’s multifunctional application potential. Future research should address these limitations, which point the way for optimizing and expanding subsequent studies.

## Conclusions

In conclusion, we have developed an epoxomicin-functionalized thermosensitive hydrogel delivery system. Maintaining the excellent biocompatibility, injectability and *in situ* gelling capability of the thermosensitive hydrogel, this system achieves localized release and precise drug regulation at the bone defect site. Experiments have shown that this system can effectively inhibit the activation of NF-κB signaling, reduce inflammatory secretion and significantly suppress osteoclast differentiation, thereby improving the bone microenvironment and promoting bone defect repair. It provides an effective local delivery strategy that integrates targeting, safety and high efficiency for the treatment of osteoporotic bone defects.

## Supplementary Material

rbag141_Supplementary_Data
