# Supplementary material for: Epoxomicin-functionalized thermosensitive hydrogel rescues bone defects in osteoporosis by the NF-κB signaling pathway
Source: Regen Biomater. 2026 Jul 15;13:rbag141. doi: 10.1093/rb/rbag141 (PMC13471993; doi:10.1093/rb/rbag141)
Supplement: Supplementary file 1 [file rbag141_supplementary_data.zip › RB-2026-078 supporting information.pdf]

SUPPORTINGH INFORMATION

Epoxomicin-Functionalized Thermosensitive Hydrogel Rescues Bone Defects in Osteoporosis by the NF-κB Signaling Pathway

Liqun Jiang[1,2,3†], Qijia Sui[1,2,3†], Chen Sun[1,2,3†], Chen Li[1,2,3], Shuai Wang[1,2,3], Zijiao Zhang[1,2,3*], Huiying Liu[1,2,3*], and Guowu Ma[1,2,3*]

1 School of Stomatology, Dalian Medical University, Dalian 116044, China.

2 Academician Laboratory of Immune and Oral Development & Regeneration, Dalian Medical University, Dalian 116044, China.

3 Dalian Medical University School and Hospital of Stomatology, Dalian 116044, China.

[†] *These authors contributed equally to this work.*

*Correspondence: zhangzj01@dmu.edu.cn (Zijiao Zhang), lhy04512000@dmu.edu.cn, lhy04512000@163.com. (Huiying Liu), mgw640242000@aliyun.com； magw@dmu.edu.cn (Guowu Ma).

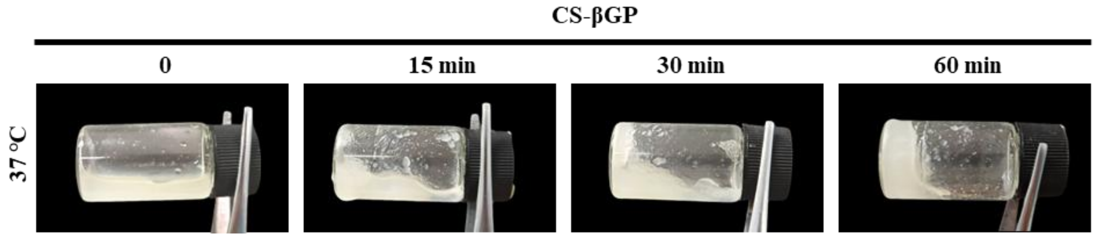

Figure S1. Gelation kinetics of CS-βGP and CS-βGP@Epoxomicin hydrogels. Sol–gel transition was monitored at 37 °C by the vial inversion method at 5, 10, 15, 30, and 60 min.

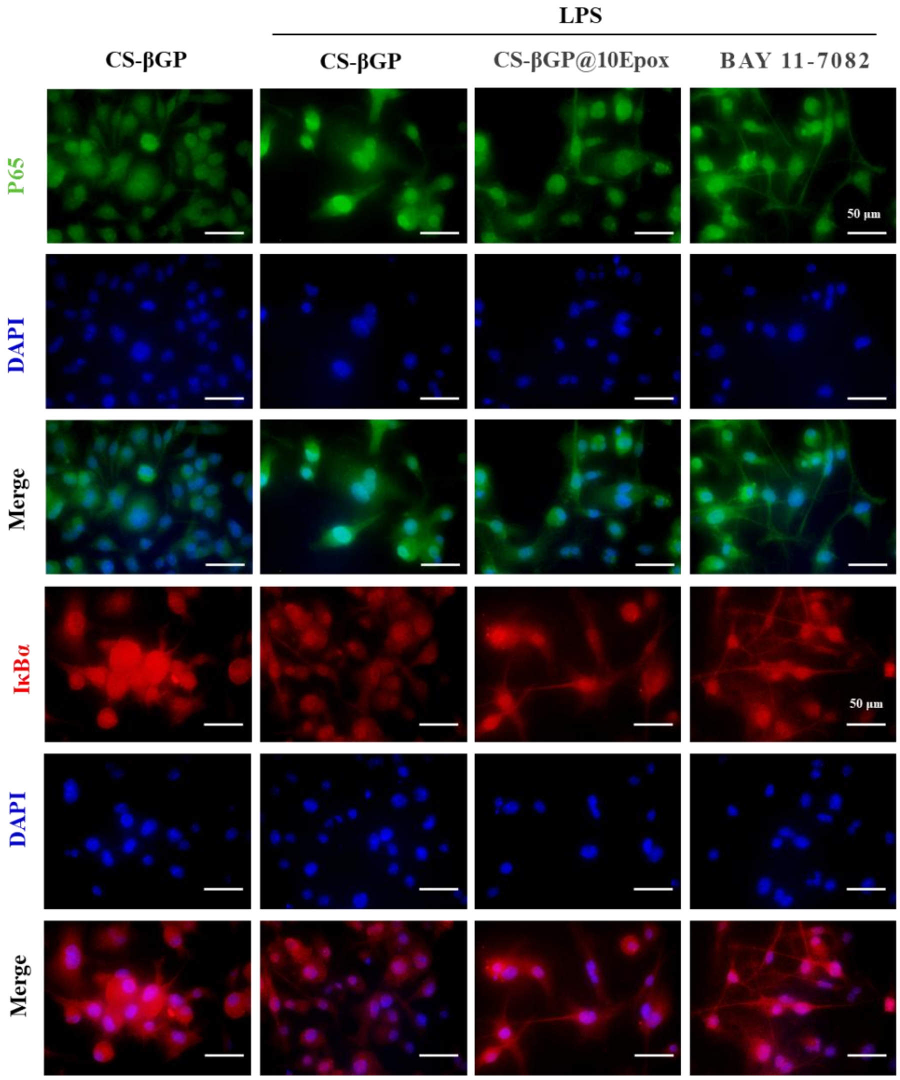

Figure S2. Immunofluorescence analysis of p65 and IκBα expression in BMMs under NF-κB inhibitor. Representative immunofluorescence images showing p65 (green)

and IκBα (red) expression in BMMs following treatment with LPS, CS-βGP@10Epoxomicin, or the NF-κB inhibitor BAY 11-7082 (1 μM) for 6 hours. Cell nuclei were counterstained with DAPI (blue).
